# Phase segregation and miscibility of TiO_*x*_ nanocomposites in Gd-doped ceria solid electrolyte material

**DOI:** 10.1107/S1600577523003636

**Published:** 2023-05-26

**Authors:** Junying Li, Prahlad K. Routh, Yuanyuan Li, Anna Plonka, Evgeniy Makagon, Igor Lubomirsky, Anatoly Frenkel

**Affiliations:** a Department of Materials Science and Chemical Engineering, Stony Brook University, Stony Brook, NY 11794, USA; b Department of Molecular Chemistry and Materials Science, Weizmann Institute of Science, Rehovot 761001, Israel; c Chemistry Division, Brookhaven National Laboratory, Upton, NY 11973, USA; ESRF – The European Synchrotron, France

**Keywords:** X-ray absorption spectroscopy, electro-chemo-mechanical effect, local structural disorder

## Abstract

Electro-chemo-mechanical (ECM) coupling refers to mechanical deformation due to electrochemically driven compositional change in a solid. An ECM actuator producing micrometre-size displacements and long-term stability at room temperature was recently reported, comprising a 20 mol% Gd-doped ceria (20GDC), a solid electrolyte membrane, placed between two working bodies made of TiO_*x*_/20GDC (Ti-GDC) nanocomposites with Ti concentration of 38 mol%. The volumetric changes originating from oxidation or reduction in the local TiO_*x*_ units are hypothesized to be the origin of mechanical deformation in the ECM actuator. Studying the Ti concentration-dependent structural changes in the Ti-GDC nanocomposites is therefore required for (i) understanding the mechanism of dimensional changes in the ECM actuator and (ii) maximizing the ECM response. Here, the systematic investigation of the local structure of the Ti and Ce ions in Ti-GDC over a broad range of Ti concentrations using synchrotron X-ray absorption spectroscopy and X-ray diffraction is reported. The main finding is that, depending on the Ti concentration, Ti atoms either form a cerium titanate or segregate into a TiO_2_ anatase-like phase. The transition region between these two regimes with Ti(IV) concentration between 19% and 57% contained strongly disordered TiO_*x*_ units dispersed in 20GDC containing Ce(III) and Ce(IV) and hence rich with oxygen vacancies. As a result, this transition region is proposed to be the most advantageous for developing ECM-active materials.

## Abstract 2

The local structure of titania–ceria composites in a Gd-doped ceria solid electrolyte material is obtained by a combination of X-ray absorption fine structure and X-ray diffraction over the entire range of Ti composition. A compositional region (between 19 and 57%) with strongly distorted TiO_6_ units and coexistence of Ce(III) and Ce(IV) was discovered, *i.e.* optimized for oxygen transport conduction and in agreement with the enhanced electro-chemo-mechanical effect previously observed in this composition range.

## 1. Introduction

Electro-chemo-mechanical (ECM) coupling (Bishop *et al.*, 2014 ▸, 2017 ▸; Swallow *et al.*, 2014 ▸; Tuller & Bishop, 2011 ▸) generates large strain and dimensional changes in nanocomposites induced by the electrochemically driven compositional changes in the solid. Although the strain induced by the ECM effect is detrimental to Li-ion batteries (Sethuraman *et al.*, 2010 ▸; Swallow *et al.*, 2014 ▸; Wang *et al.*, 2015 ▸), the macroscopic strain resulting from the compositional changes across the device is attractive for an actuation mechanism. Swallow *et al.* (2017 ▸) demonstrated the first high-temperature (550°C), low-voltage ECM actuator. The induced strain was due to the mechanical response in Pr_*x*_Ce_1–*x*_O_2–δ_ (PCO) (δ is oxygen deficiency) films as a result of applied bias (Swallow *et al.*, 2017 ▸). However, the displacements produced were too small for practical use. The ECM-based actuator with a sandwich-like structure was recently fabricated by Makagon *et al.* (2021 ▸). It is able to produce micrometre-size displacements and long-term stability at room temperature. The key innovation behind this actuator that allowed it to operate at room temperature is the use of the active layers (ActLs) made of the metal oxide/20 mol% Gd-doped ceria (20GDC) nanocomposites, serving as working bodies (WBs) (Makagon *et al.*, 2021 ▸, 2022 ▸) and separated by a micrometre-thick ionic conductor (IC) made of 20GDC, serving as a solid electrolyte (SE). After applying bias, oxygen ion transport through the IC electrolyte and the WBs undergo changes in volume upon oxidation or reduction, leading to the expansion in one ECM ActL and contraction in the other ECM ActL, inducing micrometre-sized displacement in the test structure (Makagon *et al.*, 2021 ▸).

Ti oxide/20GDC (Makagon *et al.*, 2022 ▸, 2021 ▸) (denoted as Ti-GDC for brevity) nanocomposites are found to be integral components functioning as WBs in ECM actuation. The local environment of the Ti species was reported to be a distorted TiO_6_ octahedron in the case of 38% Ti in Ti-GDC (Li *et al.*, 2021 ▸). In addition, the dimensional changes under an external electric field were shown to be related to the ordering/dis­ordering structural changes of the TiO_6_ octahedron (Li *et al.*, 2021 ▸). To maximize the ECM response, we hypothesize that the formation of local TiO_*x*_ units should play an important role. Predicting the best composition for the ECM effect is difficult because Ti may incorporate in GDC differently, depending on the Ti concentration. Ti can either substitute for Ce atoms in the GDC lattice, form cerium titanates (*e.g.* Ce_2_Ti_2_O_7_, Ce_2_TiO_5_, Ce_4_Ti_9_O_24_, CeTiO_4_ and CeTi_2_O_6_) (Preuss & Gruehn, 1994 ▸; Bazuev *et al.*, 1976 ▸; Otsuka-Yao-Matsuo *et al.*, 2004 ▸; Gionco *et al.*, 2013 ▸) or segregate into titania-rich phases. Therefore, studying the concentration-dependent structural changes in the Ti-GDC nanocomposites is essential for understanding and maximizing the mechanical deformation in the ECM actuator, which until now was designed exclusively by an Edisonian approach.

Although the local structure of Ti-GDC has not been systematically investigated to the best of our knowledge, the CeO_2_–TiO_2_ system has been studied in the past to some extent because of its promising applications as catalysts (Luo *et al.*, 2015 ▸), ferroelectric ceramics (Gao *et al.*, 2015 ▸) and electrodes (Kadhim *et al.*, 2021 ▸). Watanabe *et al.* (2009 ▸) characterized the crystalline phase and crystallite sizes of nanocrystalline TiO_2_–CeO_2_ mixed oxides (Ti_*x*_Ce_1–*x*_O_2_) by X-ray diffraction (XRD) and found that introducing a small amount of Ce (*x* > 0.9) into TiO_2_ caused a structural distortion of the anatase phase. In contrast, introducing a small amount of Ti (*x* < 0.3) into CeO_2_ caused a structural distortion of cubic fluorite phase (Watanabe *et al.*, 2009 ▸). However, when the fraction of TiO_2_ was in the range between 0.5 and 0.7, their crystalline phase and crystallite sizes were not reported by XRD likely due to the too small sizes of the crystallites and enhanced structural disorder. The formation of Ce_2_Ti_2_O_7_, containing ions of eightfold-coordinated Ce and sixfold-coordinated Ti, was detected by Raman spectroscopy and optical absorption in CeO_2_–TiO_2_ with 50 mol% CeO_2_ (Gionco *et al.*, 2013 ▸). Because the tools for detecting and interpreting structural and compositional details on nanoscale (with dimensions less than ∼3–5 nm) metal oxides are limited, X-ray absorption spectroscopy (XAS) stands out as the premier technique for this purpose (Rehr & Albers, 2000 ▸; Sayers *et al.*, 1971 ▸; Farges *et al.*, 1997 ▸; Frenkel *et al.*, 2002 ▸). X-ray absorption near-edge structure (XANES) is particularly sensitive to the local geometry of metal centers, providing information about the oxidation states and local symmetry, and not biased against structural disorder or hampered by nanoscale dimensions of materials (Rehr *et al.*, 1992 ▸; Rehr & Albers, 2000 ▸; Sayers *et al.*, 1971 ▸; Srivastava & Nigam, 1973 ▸; Ankudinov *et al.*, 2002 ▸). The pre-edge features in the XANES spectra in many cases contain key information about the electronic and structural properties of the local environment of metal oxides (Yamamoto, 2008 ▸; Srivastava & Nigam, 1973 ▸). Taking the Ti *K*-edge pre-edge features in the XANES spectra as an example, as shown by Farges *et al.* (1997 ▸), the pre-edge energy positions and signal intensities are well separated for fourfold-, fivefold- and sixfold-coordinated Ti for the Ti^4+^ species, although these results were obtained for standard compounds only. Theoretical modeling explained some of these trends and attributed the intensity of one of the pre-edge peaks to the square of the displacement of Ti atoms from the oxygen octahedral center (Poumellec *et al.*, 1991 ▸; Kraizman *et al.*, 1995 ▸). XAS has not been used for a systematic study of CeO_2_–TiO_2_ mixtures, although results obtained for several compositions of these mixtures were reported (Kityakarn *et al.*, 2013 ▸).

The objective of this study is to measure and understand the local structural environment around Ti in Ti-GDC over the broad Ti concentration range. For that, X-ray absorption spectroscopy was employed at both the Ti *K*-edge and Ce *L*_3_-edge. Synchrotron-based X-ray diffraction was used as a complementary method to investigate the long-range structure of Ti-GDC at the same concentrations. To express the series of spectral changes in terms of the number of unique species present in the samples, principal component analysis of XANES spectra was used. As a result of the combined XAS, XRD and theoretical XANES modeling, we proposed the structures of the Ti-GDC system in each concentration region and identified the range of concentrations in which the strongest ECM effect is expected.

## 2. Experiment and data analysis

Nanocomposite thin films were fabricated according to the protocol developed by Makagon *et al.* (2021 ▸). In brief, Ti-GDC samples with various Ti concentrations were deposited by a magnetron co-sputtering method on SiO_2_ substrates (around 280 µm) with a 100 nm Al adhesion layer. The samples are listed in Table S1 of the supporting information and are denoted as *x*% Ti-GDC, where *x* is the concentration of Ti. The deposition conditions are given in Table S1. XRD patterns of Ti-GDC samples were collected at beamline 28-ID-2 (λ = 0.18456 Å), National Synchrotron Light Source II (NSLS-II), Brookhaven National Laboratory, USA. XAFS data were collected at the Ti *K*-edge (4966 eV). X-ray absorption spectra of the Ti-GDC films were measured at beamline 8-BM of NSLS-II using a Si(111) double-crystal monochromator. The Ce *L*_3_-edge was measured at beamline 4-3, Stanford Synchrotron Radiation Lightsource (SSRL), using a liquid-nitro­gen-cooled Si(111) double-crystal monochromator. All spectra were collected in fluorescence mode. The raw XAFS data were analyzed utilizing the *Athena* and *Artemis* interfaces of the *Demeter* software package (Ravel & Newville, 2005 ▸). The spectra were energy-aligned, merged and edge-step normalized. *FEFF* software (Rehr *et al.*, 2009 ▸; Rehr & Albers, 2000 ▸) was utilized to calculate the theoretical XANES spectra based on a specific structural model. Titanium oxide (III), Ti_2_O_3_, was chosen as a standard to optimize the non-structural parameters.

## 3. Results and discussion

**Figure 1:**
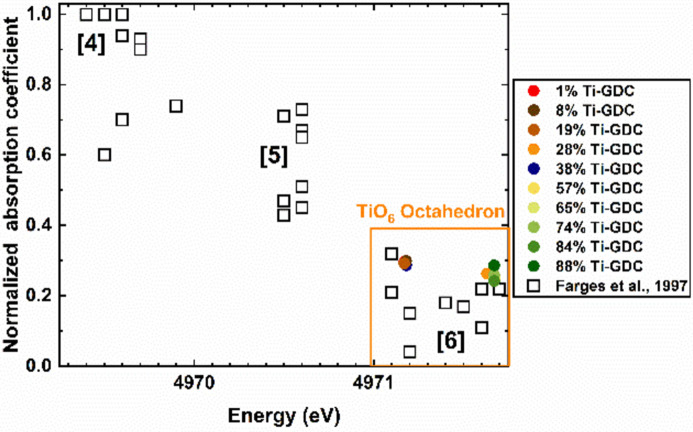
Normalized peak height versus energy position for Ti *K*-pre-edge features in XANES spectra of TiO_*x*_ units in different materials. There are three domains for fourfold-, fivefold- and sixfold-coordinated Ti {reproduced with permission from Farges *et al.* (1997) ▸ [Copyright 1997 American Physical Society]}. Colored symbols correspond to the Ti-GDC samples with different Ti concentrations. 38% Ti-GDC is reproduced from Li *et al.* (2021 ▸) with permission of the International Union of Crystallography.

For the Ti *K*-edge XANES spectra of all Ti-GDC samples, as shown in Fig. S1 of the supporting information, the rising-edge positions are close to that of TiO_2_, suggesting that the oxidation state of Ti in all Ti-GDC samples is close to Ti^4+^. The pre-edge feature A corresponds to the 1*s* to 3*d* transition in Ti and can be used for detecting and quantifying its off-center displacement (Frenkel *et al.*, 2005 ▸, 2007 ▸; Shanthakumar *et al.*, 2006 ▸). It appears, based on the calibration method developed by Farges *et al.* (1997 ▸), that, for all samples studied in Ti-GDC films with various Ti concentrations, Ti^4+^ predominantly coordinates with six oxygen atoms, as shown in Fig. 1 ▸. This conclusion and the caveats of the Farges’ analysis and its applicability for nanoscale, non-bulk-like Ti–O compounds will be discussed below. In addition, for the Ce *L*_3_-edge XANES spectra of all Ti-GDC samples, as shown in Fig. S2, the rising-edge positions are close to that of 20GDC, suggesting that the oxidation states of Ce in all Ti-GDC samples are dominated by Ce^4+^.

**Figure 2:**
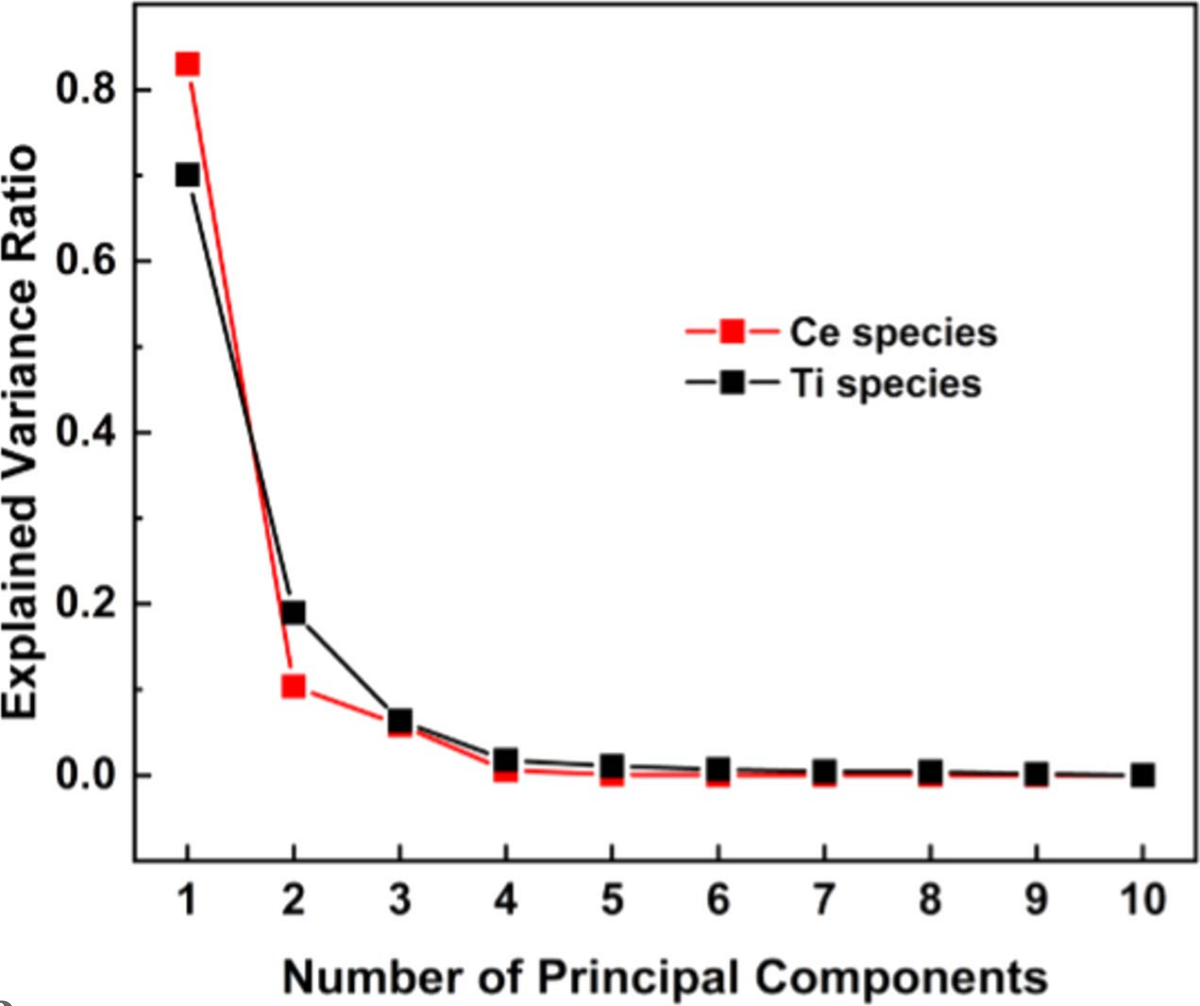
PCA scree plot for Ti species (black) for all Ti-GDC samples and Ce species (red) for all Ti-GDC and 20GDC samples.

The variations in structural features can arise from either of the two possibilities. First, at each Ti fraction, a unique species with, correspondingly, unique spectral features is formed. Alternatively, a smaller number of species are formed over the entire concentration range, and the spectral changes reflect the differences in the mixing fractions of those few species. To group the samples based on the similarity of their spectral features, we employed principal component analysis (PCA) and obtained the number of groups contributing to this series of experimental spectra (Fay *et al.*, 1992 ▸). PCA is a multivariate analysis tool, which reduces the dimensionality of the dataset while preserving its covariance (Jolliffe & Cadima, 2016 ▸). PCA was applied on Ti *K*-edge and Ce *L*_3_-edge XANES spectra for all Ti-GDC samples (explained in Section S3). PCA scree plots for Ti (black) and Ce (red) species are shown in Fig. 2 ▸, indicating that the maximum number of sub-groups for both Ti and Ce species is three.

**Figure 3:**
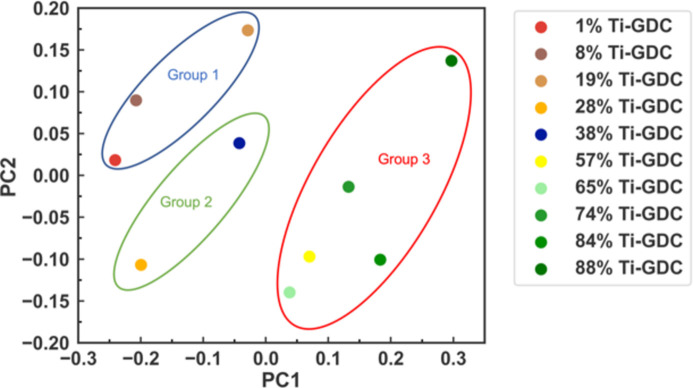
Two-dimensional principal subspace for Ti species. The species are grouped based on the similarity scores shown in Fig. S3.

The two-dimensional principal subspace (Fig. 3 ▸) can be used to group the samples based on the cosine similarity scores (Fig. S3) obtained by analyzing Ti *K*-edge XANES spectra. Cosine similarity scores are defined as cos(θ) = 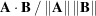 , where **A** (or **B**) refer to the vectors from the origin to the point of each sample in the principal subspace. Based on their cosine similarity score, XANES spectra are divided into three groups for Ti species: region I (1%, 8% and 19% Ti-GDC), transition region [28% Ti-GDC, 38% Ti-GDC reproduced from Li *et al.* (2021 ▸)] and region II (57%, 65%, 74%, 84% and 88% Ti-GDC). This grouping is consistent with the results obtained by Ce species, as shown in Figs. S4 and S5.

**Figure 4:**
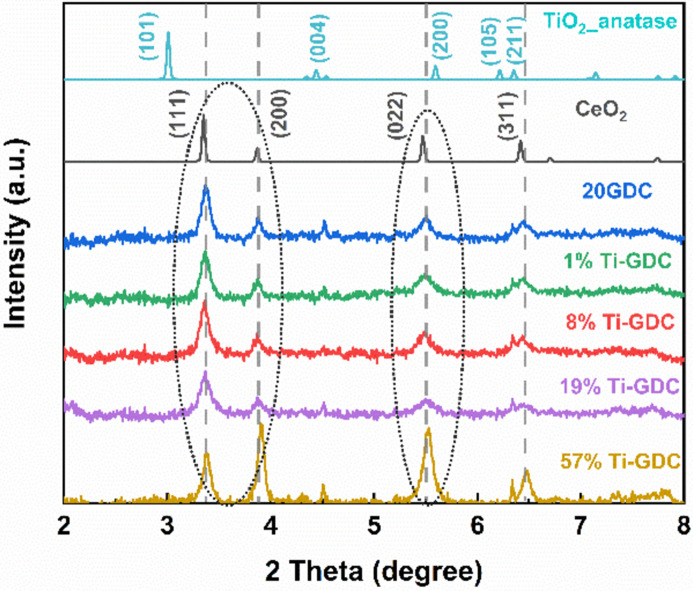
XRD pattern of the 20GDC, 1%, 8%, 19% (region I) and 57% Ti-GDC (region II) with reference fluorite CeO_2_ and anatase TiO_2_ calculated by *VESTA* software (Momma & Izumi, 2011 ▸).

To gain additional insight into the structural differences between the groups, we examined the XRD data. As shown in Fig. 4 ▸, it is apparent that fluorite CeO_2_ is found in region I (1%, 8% and 19% Ti-GDC) and region II (57% Ti-GDC) via a series of (111), (200), (022) and (311) diffraction peaks. However, as shown in Fig. 4 ▸, the intensity of the (111) diffraction peak decreased, while the intensity of the (200) and (022) diffraction peaks increased in 57% Ti-GDC, indicating that interaction of Ti oxide with 20GDC distorted the CeO_2_ lattice. The rest of the samples do not show any sharp peak features, as shown in Fig. S6, indicating that either ultra-small nanocrystals or amorphous structure would be formed in these Ti-GDC samples. In the following sections, we will describe the local structures of the samples in each region.

### 3.1. Region I

**Figure 5:**
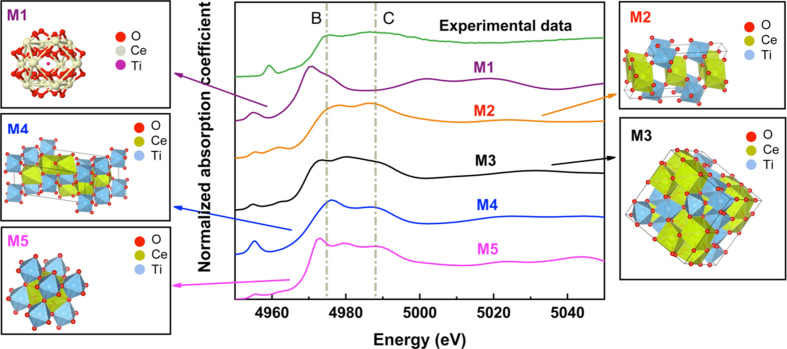
Experimental XANES spectra of Ti atoms in 8% Ti-GDC and simulated XANES spectra of Ti atoms in M1 (Ti replaces Ce in CeO_2_), M2 (CeTi_2_O_6_), M3 (Ce_2_Ti_2_O_7_), M4 (Ce_2_TiO_5_) and M5 (CeTiO_3_). The structure files for M1–M5 were obtained from the Materials Project database (Petousis *et al.*, 2017 ▸; Munro *et al.*, 2020 ▸; Patel *et al.*, 2019 ▸; Jain *et al.*, 2013 ▸) and rendered using *VESTA* (Momma & Izumi, 2011 ▸).

Based on the PCA-based grouping, region I contains 1%, 8% and 19% Ti-GDC samples. Several models can be proposed for the local environments of Ti. One such model (M1) is substitutional, in which Ti replaces the Ce atom in the cubic fluorite structure of CeO_2_. Another model is Ti forming local TiO_*x*_-type structures, that correspond to region 3 (see below) and, hence, are excluded from consideration in the region I. The other models correspond to the known Ce–Ti–O stoichiometries with TiO_6_ octahedra, namely M2 (CeTi_2_O_6_), M3 (Ce_2_Ti_2_O_7_), M4 (Ce_2_TiO_5_) and M5 (CeTiO_3_), as shown in Fig. 5 ▸. The key difference in the local structures of Ti and Ce atom environments in these phases is as follows. In M1, Ti is surrounded by eight oxygen atoms in a cubic TiO_8_ unit. Ti is bonded to six oxygen atoms to form corner-sharing TiO_6_ octahedra in CeTi_2_O_6_ (M2), and to form corner-sharing TiO_6_ octahedra with six equivalent TiO_6_ octahedra and edge-sharing with six equivalent CeO_8_ in Ce_2_Ti_2_O_7_ (M3). In Ce_2_TiO_5_ (M4), Ce is bonded to six oxygen atoms. In CeTiO_3_ (M5), Ce is bonded to twelve oxygen atoms to form CeO_1__2_, and Ti is coordinated with six oxygen atoms.

We expect that the changes in the local environments between models M1–M5 should be reflected in the corresponding changes in their Ti *K*-edge XANES spectra. EXAFS data at the Ti *K*-edge (Fig. S7) were theoretically analyzed. The fitting model included the Ti–O scattering path, and its parameters were: coordination number, correction to the bond length, its variance and the energy origin correction. The amplitude reduction factor was obtained by analysis of bulk anatase EXAFS data and fixed in the fitting of all Ti-GDC samples, which is a standard procedure. The data and fits are shown in Fig. S8. The fitting results are given in Table S2, which demonstrates that, in agreement with the XANES results already reported in our manuscript, the coordination number of Ti–O bonds is not consistent with the substitutional model (for which the Ti–O coordination number should be equal to 8). A precise model of the Ti environment is not easy to glean from EXAFS analysis, due to the uncertainty in the amplitude factor obtained by EXAFS (0.6 ± 0.2) and the limited *k*-range (only the 2–9 Å^−1^ *k*-range could be used). In order to identify the possible structures of the Ti species, we performed theoretical XANES modeling. The experimental XANES spectrum of Ti_2_O_3_, as shown in Fig. S9, was chosen as a standard to optimize the modeling (using *FEFF* code) parameters. The theoretical spectrum of Ti_2_O_3_ contains two key features (B and C) seen also in the experimental spectrum. Fig. 5 ▸ shows that the theoretical spectra of CeTi_2_O_6_ and Ce_2_TiO_5_ are the best models for reproducing the two key features (B and C) of the experimental data. However, as shown by the Ce *L*_3_-edge spectra [Fig. S2(*b*)], the local structure of Ce is similar to that in 20GDC, implying that the Ce ions in all Ti-GDC samples in region I are coordinated with eight oxygen atoms, as opposed to the six oxygen atoms in Ce_2_TiO_5_. Therefore, the CeTi_2_O_6_ structure, which contains a similar Ce environment to that of 20GDC, wins (our PCA analysis for Ce *L*_3_-edge XANES is inconsistent with the mixed Ce environment in region 1, as shown in Figs. S4 and S5), and we identify it as the prevailing model of the local structure of Ti in region I.

### 3.2. Region II

**Figure 6:**
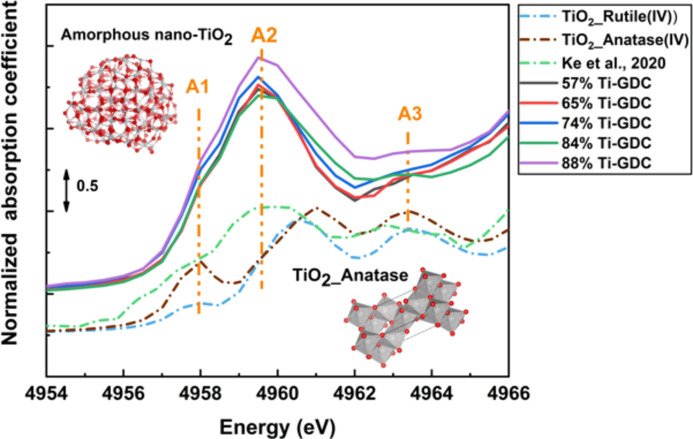
Ti *K*-pre-edge features for Ti-GDC samples in region II. The distorted anatase Ti *K*-edge spectrum and reverse Monte Carlo generated amorphous nano-TiO_2_ structure are reproduced with permission from Ke *et al.* (2020) ▸ [Copyright 2020 American Chemical Society] and Zhang *et al.* (2008) ▸ [Copyright 2008 American Physical Society], respectively. The structure of TiO_2__anatase was generated using information from the Materials Project database (Petousis *et al.*, 2017 ▸; Munro *et al.*, 2020 ▸; Patel *et al.*, 2019 ▸; Jain *et al.*, 2013 ▸).

Region II contains 57%, 65%, 74%, 84% and 88% Ti-GDC samples. According to Fig. 1 ▸, the local environment of Ti is octahedral. In contrast to the pre-edge features in the Ti-GDC samples in region I (Fig. S1), the dominant feature in the pre-edge region is the split into A1 and A2 features, as shown in Fig. 6 ▸. The positions of the A1, A2 and A3 features are similar to the anatase phase reported by Ke *et al.* (2020 ▸), but the intensity of these features is much higher for the Ti-GDC samples in our work. The intensity of the pre-edge features is weak in centrosymmetric environments (Luca *et al.*, 1998 ▸), and increases as the environment is distorted. Empirical approaches have been used by Luca *et al.* (1998 ▸) to establish correlations between the Ti *K* pre-edge transitions in anatase-TiO_2_, indicating that the intensity ratio (*I*_A1_/*I*_A2_) of the Ti *K*-pre-edge feature A1 to A2 transitions [equivalent peaks of Luca *et al.* (1998 ▸) are labeled as A2 and A3, respectively] increases as particle size decreases, as well as the surface-area-to-volume ratio of the particles increasing. Farges *et al.* (1996 ▸) demonstrated that the A1 peak is due to the pentacoordinated Ti atoms. Zhang *et al.* (2008 ▸) simulated the amorphous nano-TiO_2_ structure by utilizing reverse Monte Carlo (RMC) and demonstrated that feature A1 was also from the distorted Ti–O octahedra. EXAFS analysis of samples in region II, as shown in Table S2, indicates that the coordination numbers decrease below 6 at high Ti concentration, although the trend is difficult to identify due to the experimental error bars. A reduction of the Ti–O coordination number with respect to the bulk analog (anatase) of 6 is expected in the case of nano-sized TiO_2_, in which the contribution of the under-coordinated Ti atoms on the surface lowers the average coordination number. This effect was also predicted by simulations (Zhang *et al.*, 2008 ▸) and observed in several experimental studies (Shkrob *et al.*, 2004 ▸; Rajh *et al.*, 2002 ▸; Chen *et al.*, 1999 ▸). It also does not contradict the conclusion based on the interpretation of the pre-edge intensity (Fig. 1 ▸) because the Farges’ method was calibrated using bulk-like, standard Ti–O compounds only. Thus, we propose that the disordered nanoscale TiO_2_ structure was formed in region II.

**Figure 7:**
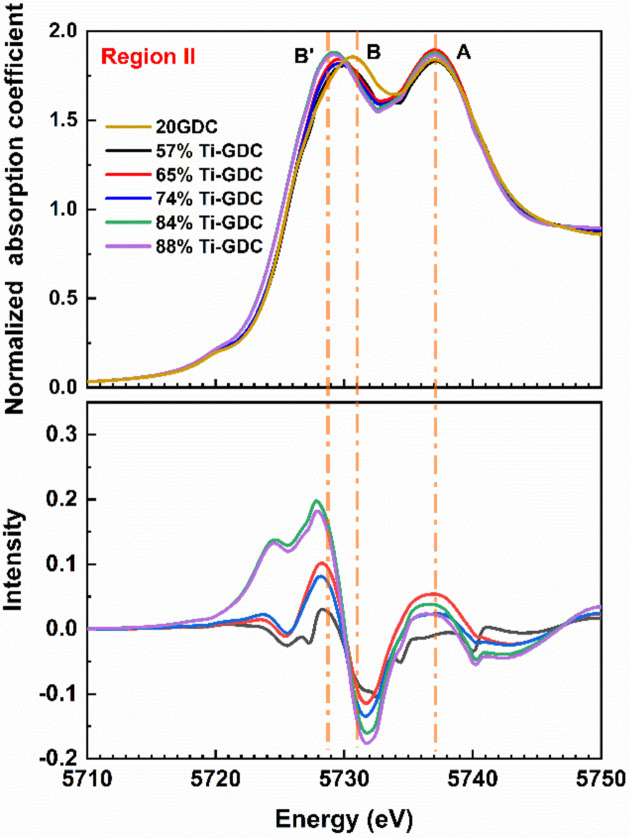
Ce *L*_3_-edge XANES spectra are shown in the top pane for the 57%, 65%, 74%, 84%, 88% Ti-GDC sample and 20GDC sample. 20GDC data have been subtracted from each spectrum. The difference between the Ti-GDC and 20GDC data is shown in the bottom pane.

As shown in Fig. 7 ▸ (top), there are two main Ce *L*_3_-edge peaks: peak A (2 *p* *f* ^0^5*d* state), which is only expected for Ce^4+^ species, and peak B (2 *p* *f* ^1^5*d* ^*^ *L* state), which originates from the charge transfer from oxygen to the Ce 4*f* orbital (Soldatov *et al.*, 1994 ▸; Kossoy *et al.*, 2013 ▸; Bianconi *et al.*, 1987 ▸). 2 *p* denotes the empty state in the 2*p* shell and *L* denotes an empty state in the neighboring oxygen orbital. Following the process described by Overbury *et al.* (1998 ▸), the 20GDC data were subtracted from each of the Ti-GDC absorption coefficients, and the differences between the Ti-GDC and GDC data are shown in Fig. 7 ▸ (bottom). With increasing Ti concentration, there is a progressive decrease in the intensity of peaks B at 5731 eV, but at the same time peak B′ is growing, corresponding to the reduction of the Ce species. Hence, both Ce^4+^ and Ce^3+^ species are present in the Ti-GDC samples in region II.

### 3.3. Transition region

**Figure 8:**
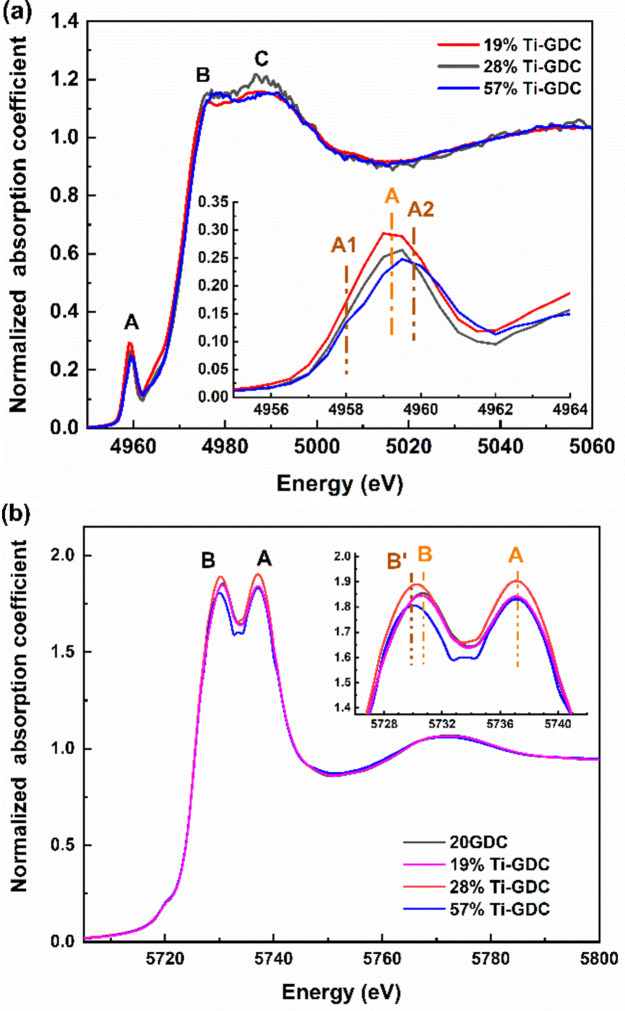
(*a*) Normalized Ti *K*-edge XANES spectra of 28% Ti-GDC and references: 19% Ti-GDC and 57% Ti-GDC. Inset: enlarged pre-edge region. (*b*) Normalized Ce *L*_3_-edge XANES spectra of 28% Ti-GDC and references: 20GDC, 19% Ti-GDC and 57% Ti-GDC. Inset: enlarged region of the main peaks.

According to the results obtained by PCA, the 28% Ti-GDC sample is not identified as either region I or region II. According to Fig. 1 ▸, the local environment of Ti is octahedral. As shown in Fig. 8 ▸(*a*), the normalized Ti *K*-pre-edge XANES spectrum of 28% Ti-GDC is close to that in the 19% Ti-GDC sample, with only one prominent peak rather than the two splitting peaks in 57% Ti-GDC samples. In addition, the intensity of the pre-edge peak A in 28% Ti-GDC is lower than that in the 19% Ti-GDC sample. Moreover, as shown in Fig. 8 ▸(*b*), the position of the peak B of the Ce *L*_3_-edge XANES spectrum of 28% Ti-GDC is between the 20GDC and the 57% Ti-GDC sample, indicating the coexistence of the Ce^4+^ and Ce^3+^ species. Therefore, the region between region I and region II is transitional between nanoscale cerium titanate structure and phase-segregated disordered titania.

Based on the combination of experimental and theoretical XANES spectroscopy of Ti *K*- and Ce *L*_3_-edges and XRD studies, we identified the range of Ti concentrations (from 28% to 88%, using multiple Ti-GDC samples with controlled Ti compositions) in which TiO_*x*_ can be detected as a separate region. This result is consistent with the previous work in which 38% Ti in Ti-GDC was found to be electro-chemo-mechanically active (Makagon *et al.*, 2021 ▸). TiO_*x*_ with locally disordered octahedral TiO_6_ units undergoes rapid oxidation and reduction during ECM coupling. However, ECM devices with the working body consisting of 100% TiO_*x*_ show immediate saturation of the ECM response due to a lack of oxygen transport (Mishuk *et al.*, 2019 ▸). Hence, the coexistence of the Ce^4+^ and Ce^3+^ species should be the key descriptor for characterizing oxygen diffusion in the ECM actuators. Based on this study we propose that other mixed TiO_2_–CeO_2_ systems within the broader range of Ti concentrations can also be ECM-active. For a search of the strongest effect in Ti-GDC composites we propose to investigate the concentrations in the transition region (between 19 and 57 mol% Ti) due to the observed strong local distortion of Ti in the TiO_6_ units (hence, capable of generating large local strains) and coexistence of Ce^4+^ and Ce^3+^ (hence, facilitating oxygen transport). Our analysis methodology developed for this material can be used for analyzing a large class of functional mixed metal oxides.

## 4. Conclusions

The volumetric changes in the two working bodies made by TiO_*x*_/20GDC (Ti-GDC) are essential to generate large mechanical deformation in the ECM actuator. Both XAS and XRD were applied to study the structural changes of Ti-GDC and clarify the local structure of Ti and Ce in Ti-GDC with various Ti concentrations. We detected three different types of local structural units at different Ti concentrations using principal component analysis of XANES spectra and identified the likely structures in each region. In regions I and II, the local structure of Ce species is similar to that in fluorite CeO_2_. Nanoscale cerium titanates were formed in region I, while in region II the disordered nano-TiO_2_ structure was found. Meanwhile, both Ce^4+^ and Ce^3+^ species are present in the Ti-GDC samples in region II. Between regions I and II, there is a transition region containing TiO_*x*_ units with strongly distorted TiO_6_ octahedra along with coexisting Ce^4+^ and Ce^3+^ species. Our results will be helpful for future studies that will aim to link the ECM and other electromechanical effects (*e.g.* electrostriction) to the local geometric and electronic properties of mixed oxides.

## Supplementary Material

Sections S1 to S6, incorporating Figures S1 to S9 and Tables S1 to S2. DOI: 10.1107/S1600577523003636/ok5092sup1.pdf
