# Supplementary material for: Phase segregation and miscibility of TiO x nanocomposites in Gd-doped ceria solid electrolyte material
Source: J Synchrotron Radiat. 2023 May 26;30(Pt 4):758–65. doi: 10.1107/S1600577523003636 (PMC10325025; doi:10.1107/S1600577523003636)
Supplement: Supplementary file 1 [file s-30-00758-sup1.pdf]

JOURNAL OF SYNCHROTRON RADIATION

Volume 30 (2023)

Supporting information for article:

Phase segregation and miscibility of TiOx nanocomposites in Gd-doped ceria solid electrolyte material

Junying Li, Prahlad K. Routh, Yuanyuan Li, Anna Plonka, Evgeniy Makagon, Igor Lubomirsky and Anatoly Frenkel

S1. Sample details

Table S1 Deposition conditions for Ti-GDC nanocomposites deposited on SiO_2 substrates with Al stress relief layer. All samples were deposited at room temperature.

Sample	Ti/(Ti+Gd+Ce) (at%)	GDC Power (W)	Ti Power (W)	O_2 flow (sccm)	$O_2/(O_2+Ar)$ (%)	Deposition time (hr)
20GDC	0	110	0	3	9	3
1% Ti-GDC	1	110	70	3	9	3
8% Ti-GDC	8	110	170	3	9	3
19% Ti-GDC	19	110	230	3	9	3
28% Ti-GDC	28	50	150	3	9	3
38% Ti-GDC	38	100	150	3	8	3
57% Ti-GDC	57	85	137	1.5	5	3
65% Ti-GDC	65	85	150	1.5	5	3
74% Ti-GDC	74	85	175	1.5	5	4
84% Ti-GDC	84	65	200	1.5	5	3
88% Ti-GDC	88	65	200	1.5	5	6

S2. Ti K-edge and Ce L_3-edge XANES of TiO_x-20GDC nanocomposites

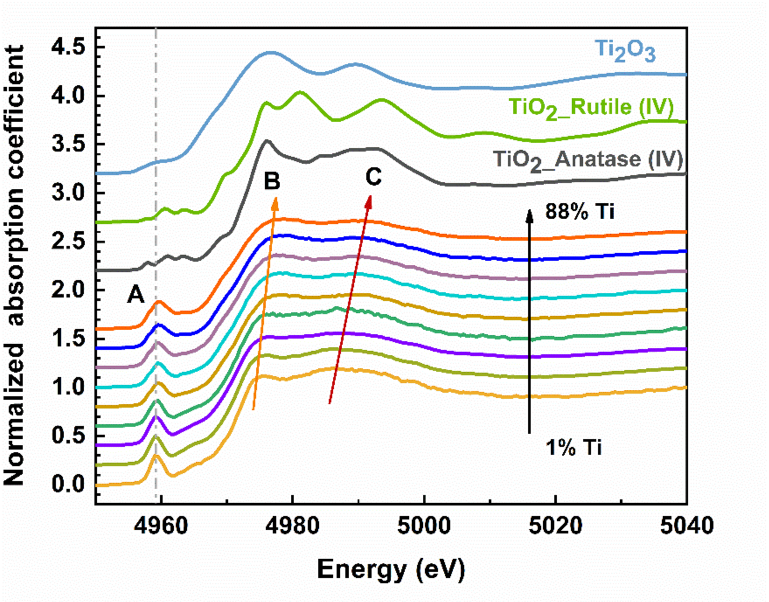

Figure S1 Normalized Ti K-edge XANES spectra of all samples with titanium oxides standards: Ti_2O_3 and TiO_2 (rutile and anatase). Spectra are vertically stacked for comparison.

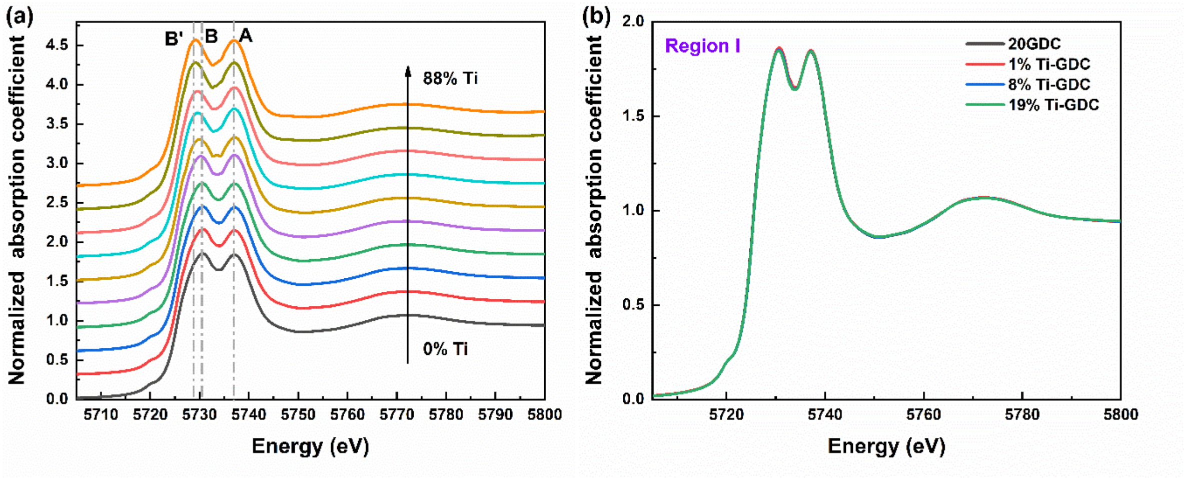

Figure S2 (a) Normalized Ce L_3-edge XANES spectra (stacked) of all samples, (b) samples in Region I only (overlayed).

S3. Dimensionality reduction and clustering of XANES using Principal Component Analysis (PCA)

XANES spectra in the energy range of E_0 - 20eV to E_0 + 80eV with E_0= 4971.23eV were selected for spectral similarity in the reduced principal space. PCA was applied to reduce the dimensionality of each XANES spectra in (energy axis) and analyze the similarity of reduced XANES spectra based on cosine similarity of vectors in principal directions. For 10 different XANES spectra, 10 eigenvalues were obtained. As shown in Fig. 2, the variance in the sample is captured by first 3 principal components, indicating the presence of 3 potential clusters. Scatterplot of first two principal components (Fig. 3) shows a visual representation of each cluster with each XANES spectra represented by only first 2 principal components. For a more objective clustering, vectors consisting of 10 principal components for each sample were used to obtained cosine similarity as defined in the main text. Figure S3, shows the cosine similarity values and nearest neighbor clusters according to these values. Similar study was carried out on Ce-L_3-edge XANES spectra as well and the results are shown in Figure S4 and Figure S5.

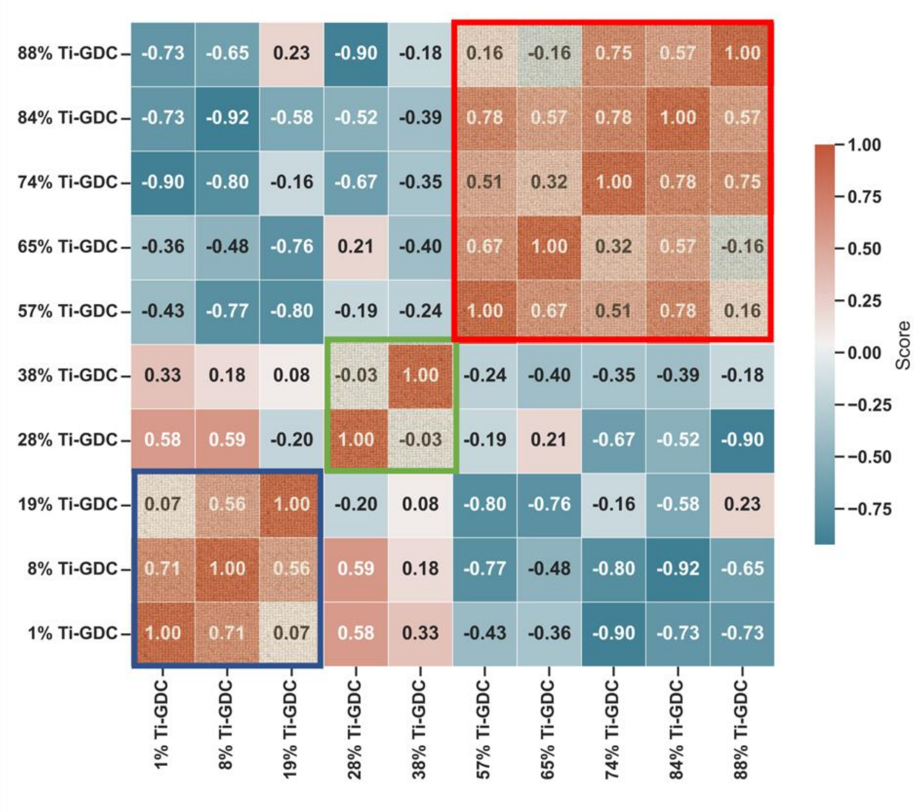

Figure S3 Heatmap for Ti species generated using the cosine similarity in principal subspace. The samples are grouped based on nearest neighbour in the similarity score.

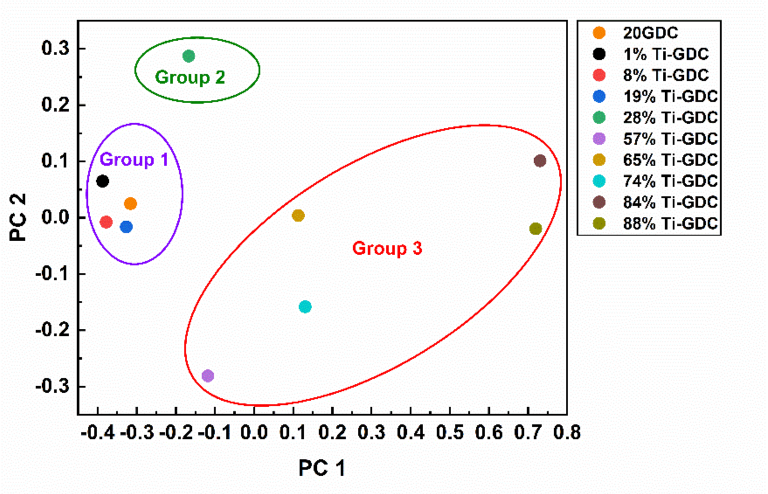

Figure S4 The two-dimensional principal subspace for Ce species. The species are grouped based on the similarity scores shown in Fig. S5.

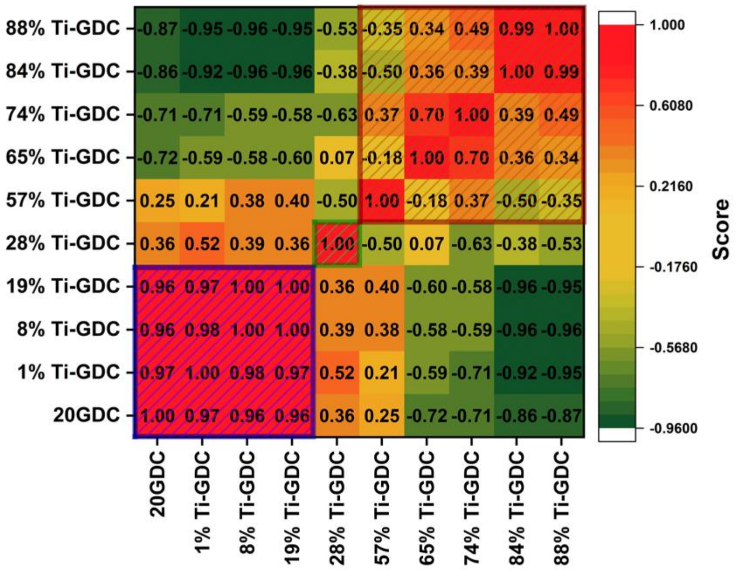

Figure S5 Heatmap for Ce species generated using the cosine similarity in principal subspace. The samples are grouped based on nearest neighbour in the similarity score.

S4. X-ray diffraction spectra of Ti-GDC

Figure 4 shows the XRD spectra for low %Ti-GDC and showing Bragg peaks corresponding to 20GDC. Remaining XRD spectra are shown below in Figure S6.

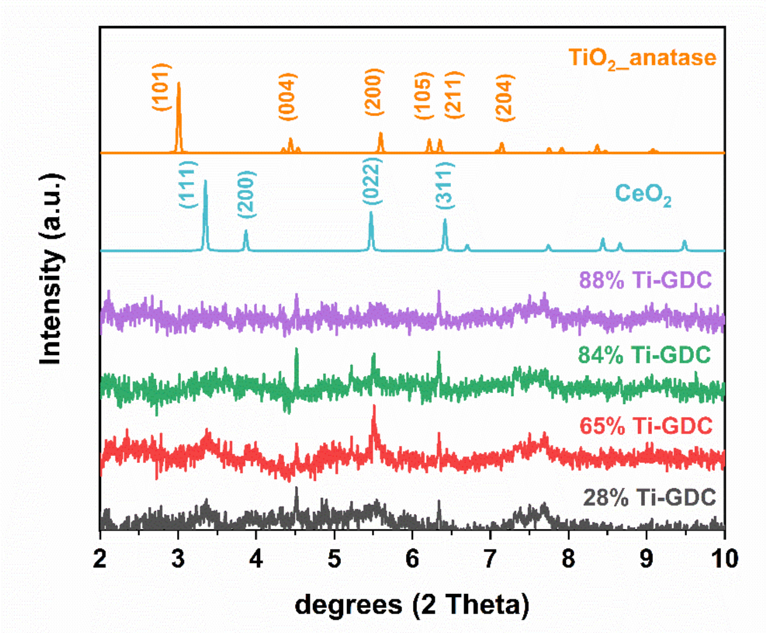

Figure S6 XRD pattern of the 28% Ti-GDC, 65% Ti-GDC, 84% Ti-GDC, and 88% Ti-GDC with CeO_2 and TiO_2 (anatase) standards.

S5. Ti K-edge EXAFS analysis of TiO_x-20GDC nanocomposites

Figure S7 shows the EXAFS spectra in k-space and r-space along with a sample consisting of TiO_2-Anatase. Figure S8 shows the data and theoretical fits in r-space for all samples analysed at Ti K-edge. Table S2 contains the best fit results.

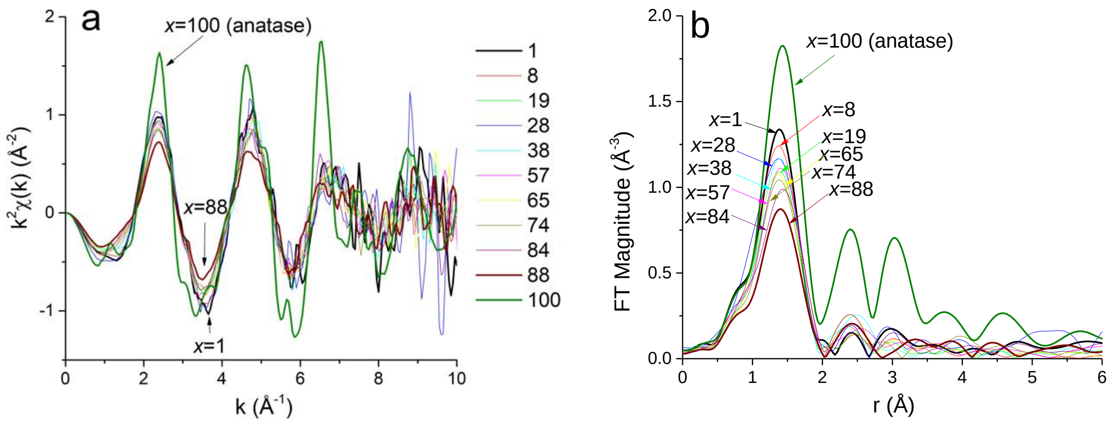

Figure S7 EXAFS data (a), k^2-weighted Ti K-edge EXAFS data in k-space, and (b) Fourier transform magnitudes of the k^2-weighted EXAFS data TiO_2 (anatase) and Ti-GDC samples.

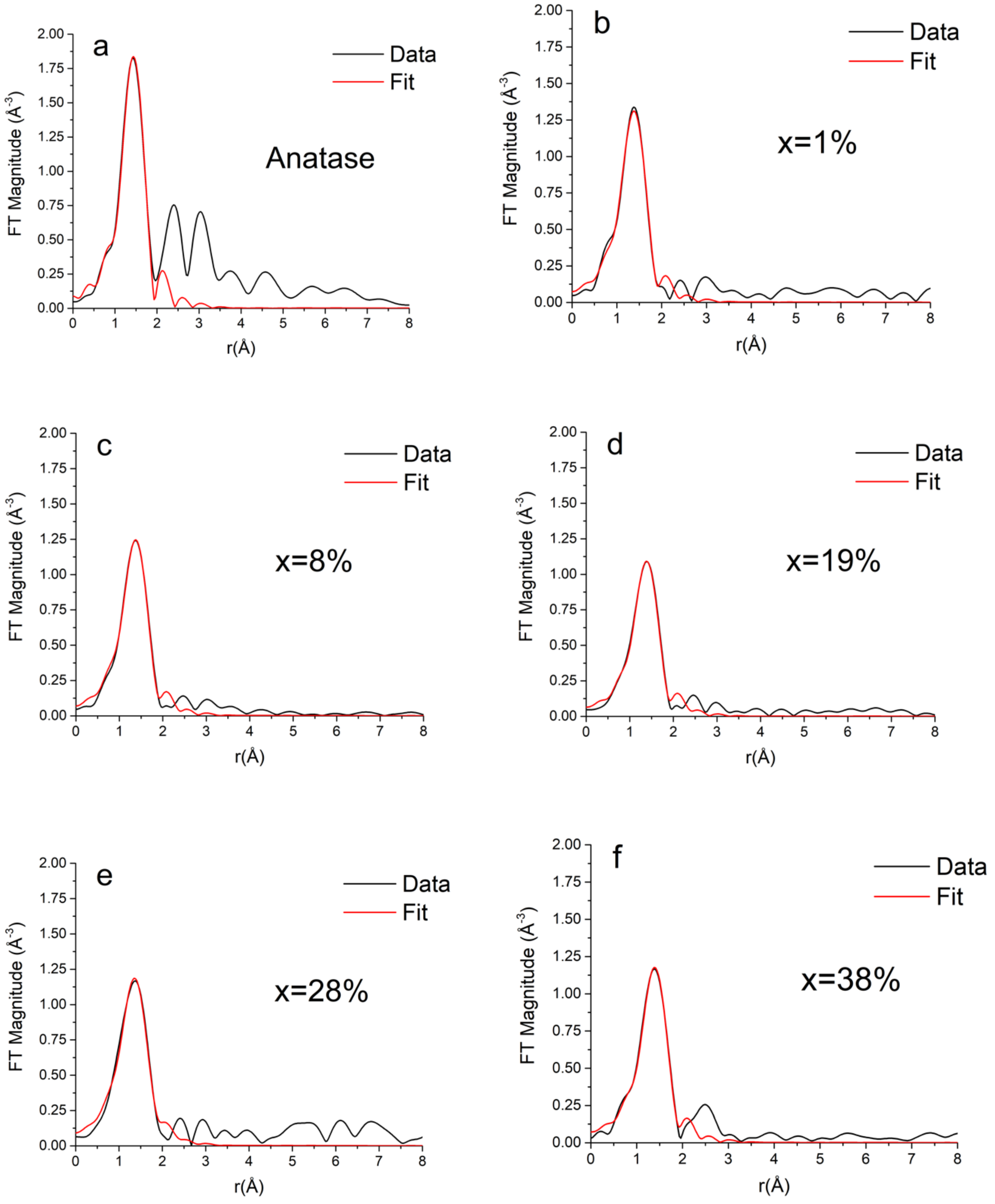

a
Anatase
b
x=1%
c
x=8%
d
x=19%
e
x=28%
f
x=38%
Data
Fit
FT Magnitude (Å^{-3})
r(Å)

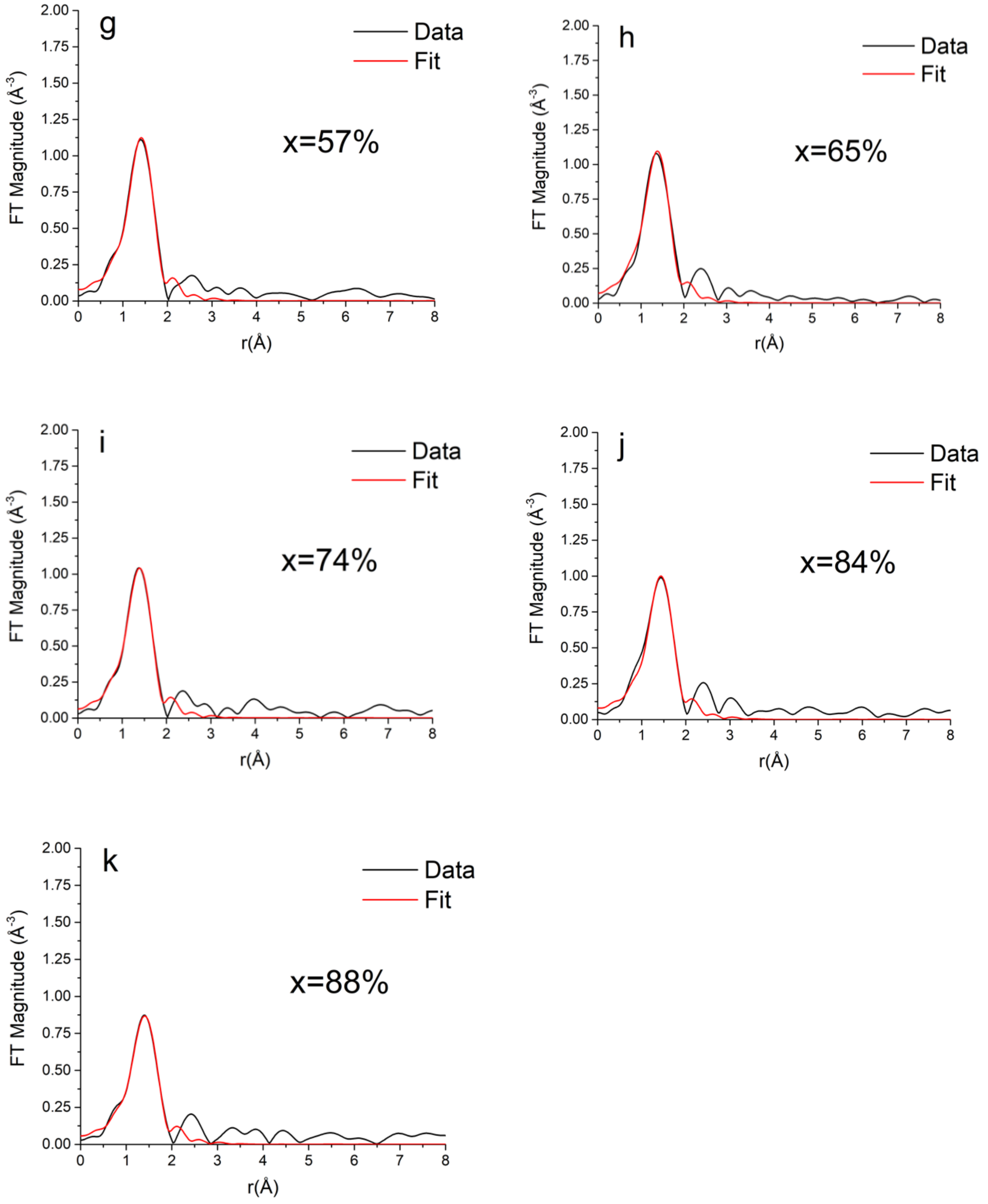

Figure S8 Fourier transform magnitudes of k^2-weighted EXAFS data and theoretical fits for the TiO_2 – anatase (a) and Ti-GDC samples (b-k).

Table S2 Fit results for the Ti-GDC samples and TiO_2 – anatase (*x*=100). The S_0^2 parameter (the passive electron reduction factor) was determined from the fit to the anatase structure to be equal to 0.6 ± 0.2 and fixed to 0.6 in the fits for the rest of the samples.

x	$N_{Ti\text{-}O}$	$R_{Ti\text{-}O}$ (Å)	$\sigma^2_{Ti\text{-}O}$ (Å^2)
1	5.3(7)	1.91(1)	0.006(2)
8	5.6(9)	1.91(2)	0.007(3)
19	4.5(8)	1.92(2)	0.006(3)
28	6.8(1.4)	1.91(2)	0.012(4)
38	4.8(1.3)	1.92(3)	0.006(4)
57	5.0(1.1)	1.94(2)	0.007(4)
65	5.6(2.1)	1.92(4)	0.010(6)
74	4.6(1.1)	1.92(2)	0.007(4)
84	4.5(1.2)	1.95(3)	0.008(5)
88	3.7(6)	1.92(3)	0.007(3)
100 (anatase)	6 (fixed)	1.95(3)	0.002(5)

S6. FEFF parameter optimization for XANES modelling

FEFF 9.9.1 was used in this study to calculate the theoretical XANES spectra. Ti_2O_3 standard and corresponding experimental and FEFF generated theoretical spectra (the structure was obtained from Inorganic Crystal Structure Database), as shown in Fig S9, were used to validate the non-structural FEFF parameters which are further used to simulate the spectra of other Ti species. S_0^2 of 0.9, where the core-hole was treated with Final State Rule (FSR), (non-screened) was used for the Ti K-edge XANES simulation. The radius of the cluster was 4 Å, the maximum number of iterations was 30, the convergence accelerator factor was 0.2. Dipole and magnetic dipole transitions were included in the calculations. Hedin-Lundquist exchange correlation potential was used for the fine structure and Dirac-Hara is used for the background function. The maximum k value of 6 Å^{-1} and the grid of 0.06 Å^{-1} were used for the XANES card in FEFF calculation.

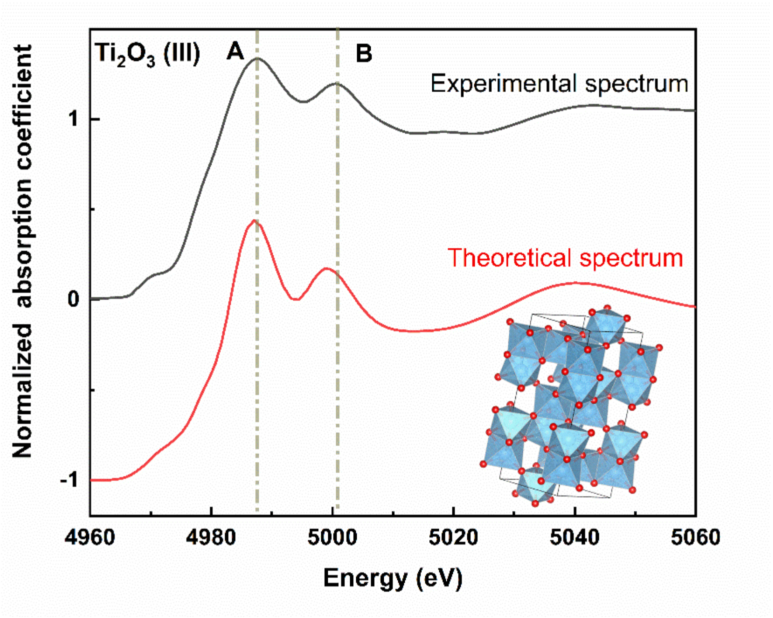

Figure S9 Experimental and simulated XANES spectra of Ti atoms in Ti_2O_3.
